# Long enduring response on teclistamab in a patient with myeloma relapse after allogeneic hematopoietic stem cell transplantation – a case report

**DOI:** 10.1007/s00277-026-06990-6

**Published:** 2026-04-07

**Authors:** P. Ebner, J. Fauser, G. Hetzenauer, S. Köck, W. Willenbacher, L. M. Rossetti, T. A. Buchwald, L. Rasche, E. Gunsilius, D. Nachbaur, D. Wolf, N. Steiner

**Affiliations:** 1 University Hospital of Internal Medicine V (Hematology and Oncology), Comprehensive Cancer Center Innsbruck (CCCI), Medical University of Innsbruck, Anichstraße, 35, Innsbruck, 6020 Austria; 2 Department of Internal Medicine II, University Hospital of Würzburg, Grombühlstraße 12, 97080 Grombühl, Würzburg, Germany; 3 Department of Nuclear Medicine, Medical University of Innsbruck, Anichstraße 35, Innsbruck, 6020 Austria

**Keywords:** Relapsed/refractory myeloma, Teclistamab, TCE after allo-HSCT, Long-enduring treatment response, Graft versus myeloma effect

## Abstract

Cellular based therapies, such as bispecific T-cell engagers (TCE), have changed the landscape of myeloma treatment, but to the present day little is known about the use of these therapies in patients with prior allogeneic hematopoietic stem cell transplantation (allo-HSCT). In this case report, we present a patient with multi-refractory multiple myeloma (MM), who relapsed after allo-HSCT and anti-SLAMF-7-CART-cell-therapy and was ultimately successfully treated with teclistamab, a BCMA-directed TCE, and we discuss theoretical considerations based on present literature about possible underlying immunological mechanisms, that might have contributed to a long enduring treatment response.

## Introduction

In relapsed/refractory (r/r) MM, treatment with TCE has become an established therapeutic option. Teclistamab was the first BCMA-directed TCE approved for r/r myeloma [1] based on the data generated in the Majes-TEC-1 study, showing a remarkable overall response rate of 63% in myeloma patients with 3 or more prior treatment lines. Currently, two additional BCMA-directed TCE and one GPRC5D-directed TCE as well as BCMA-directed CAR-T-cells are approved, changing the treatment landscape of MM. Before their implementation, allo-HSCT represented a viable treatment option for a few multirefractory, but young and fit patients, leading to long-term disease control in about 25% [2]. However, many patients experience disease relapse after allo-HSCT and conceptually, the subsequent use of TCE is a promising treatment option, even though the induction of graft-versus-host-disease is a potential risk. To the best of our knowledge only one case report of teclistamab after allo-HSCT has been published so far, with a short follow-up and limited extramedullary involvement [3].

In this article, we present the case of a heavily pretreated patient with multi-refractory MM, who relapsed after allo-HSCT and anti-SLAMF-7-CAR-T-cell-therapy, and was successfully treated with teclistamab.

## Case report

**Fig. 1:**
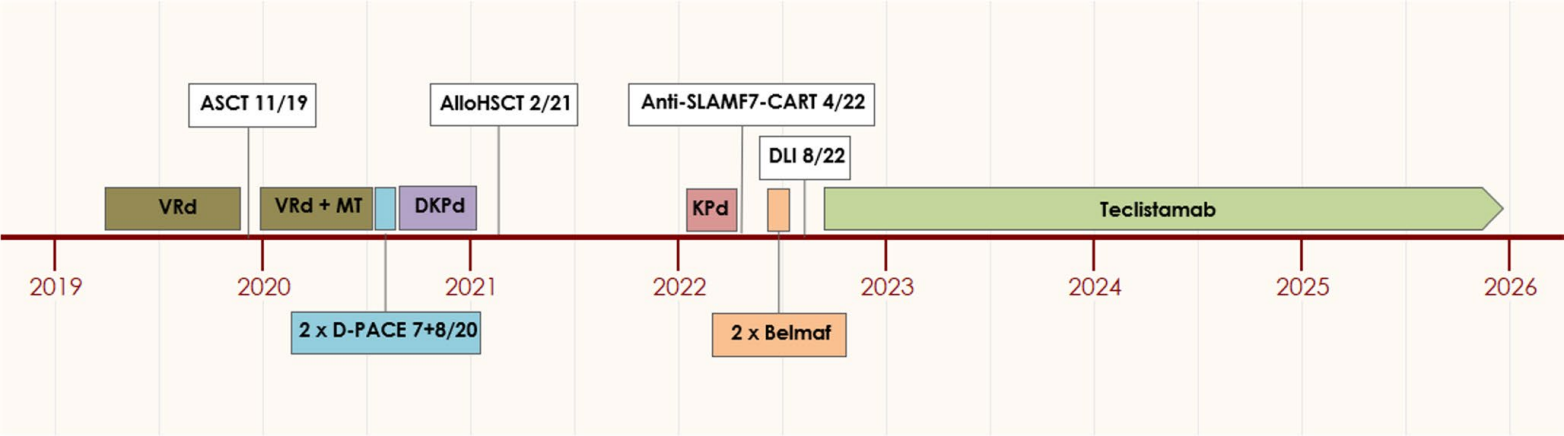
Overall treatment history with following treatment lines. 1^st^ line: 5 cycles VRd induction, ASCT, 2 cycles VRd consolidation, lenalidomide MT. 2^nd^ line: 2 cycles D-PACE. 3^rd^ line: DKPd. 4^th^ line: Allo-HSCT. 5^th^ line: KPd bridging + CART. 6^th^ line: 2 cycles belmaf. 7^th^ line: teclistamab. V = bortezomib, R = lenalidomide, d = dexamethasone, MT = maintenance therapy, D = daratumumab, K = carfilzomib, P = pomalidomide, belmaf = belantamab mafodotin, DLI = donor lymphocyte infusion, D-PACE = dexamethasone, cisplatin, doxorubicin, cyclophosphamide and etoposide

**Fig. 2:**
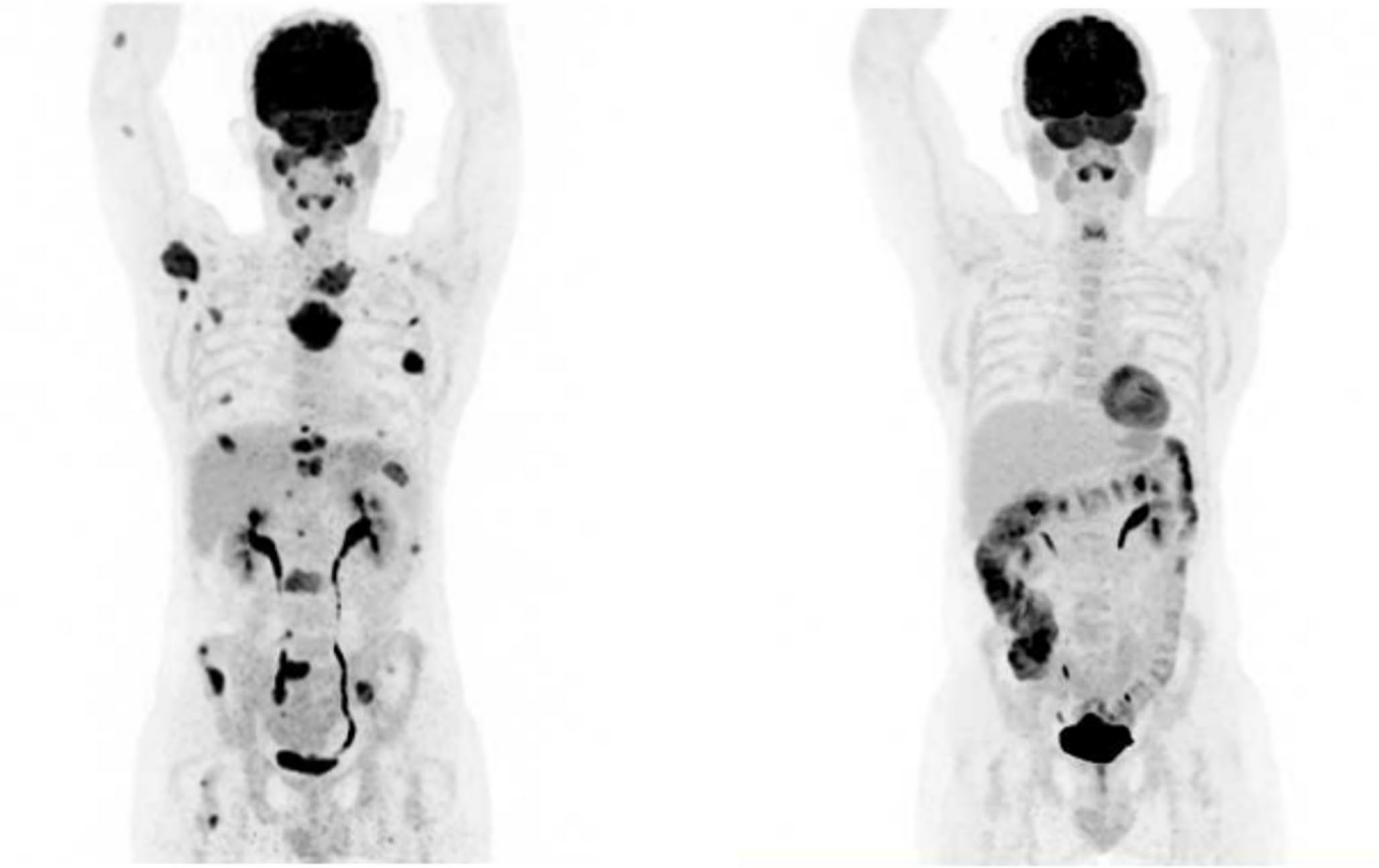
PET-CT. *Left*: 09/2022 before teclistamab was started. It shows disseminated bone and extramedullar myeloma lesions. *Right*: most recent follow-up 09/2025. It shows no pathologic tracer uptake, therefore resulting in an mCR

A male patient without relevant comorbidities was diagnosed with IgG kappa MM at the age of 43 years in May 2019. He initially presented with a pathological fracture of the right clavicula and histology showed plasma cell infiltration with a standard risk cytogenetic. In addition to multiple bone lesions, extramedullary disease was present in the left ethmoidal cells. Despite extensive bone and extramedullary manifestations, paraprotein serum levels were only slightly elevated and a random bone marrow biopsy did not show clonal plasma cell infiltration. Risk stratification revealed stage I ISS/R-ISS. First line treatment with bortezomib, lenalidomide and dexamethason (VRd) induction, autologous stem cell transplantation, VRd consolidation and subsequent lenalidomide maintenance was performed. Best response was a serologic very good partial response and a partial metabolic remission (PET-CT). In July 2020 early and rapid disease progression occurred. Two cycles of dexamethasone, cisplatin, doxorubicin, cyclophosphamide and etoposide did not show relevant effect, as well as the next treatment regime with daratumumab, carfilzomib, pomalidomide and dexamethasone (Jasiliec et al. [4]). At this point we proceeded to allo-HSCT, which was performed with a fully matched sibling donor in February 2021 (conditioning therapy: fludarabine, busulfan, melphalan, immunosuppression: cyclosporin a, mycophenolate mofetil), resulting in a partial response. No major complications occurred and the patient did not show graft versus host disease (GvHD), even after tapering immunosuppression. Disease relapse was observed one year after allo-HSCT. We then were able to enroll the still very fit patient into the CARAMBA trials (SLAMF7-directed CAR-T-cells) in collaboration with the University Hospital of Würzburg, Germany. After a bridging therapy with carfilzomib, pomalidomide and dexamethasone, CAR-T-cell infusion was administered in April 2022. No CRS or ICANS occurred, but disease progression was observed already two months after CAR-T-cell-therapy. A following individual therapy approach with the BCMA-directed antibody drug conjugate belantamab mafodotin in combination with pomalidomide and dexamethasone failed, as well as donor lymphocyte infusion (DLI). Despite the heavy pre-treatment (see also Fig. 1) the patient still showed a good condition and we enrolled him into a named patient program for teclistamab. In September 2022, step-up dosing proceeded without relevant complications and teclistamab was finally administered in a weekly standard. Soon, serological and metabolic response was observed, with PET CT showing a metabolic complete remission (mCR) (Fig. 2). Notably, there were no signs of GvHD and a full donor chimerism was documented. The most relevant adverse events were recurrent infections, which were reduced in frequency and severity by immunoglobulin substitution. With persistent mCR, treatment intervals were extended to biweekly and lately even to 4-weekly. The most recent clinical checkup in December 2025 again revealed an excellent condition of the patient and no signs of GvHD.

## Discussion

This case report impressively demonstrates the potency of teclistamab in r/r myeloma after allo-HSCT. The immunologic mechanisms leading to the excellent myeloma control without induction of GvHD are presumed to be complex and very limited clinical data is available regarding the use of TCE after allo-HSCT. We found one similar case of myeloma treatment published, which provides a very limited follow-up time of 34 weeks.^3^ In the following, we aim to discuss some considerations regarding immunological mechanisms in this setting. Since no corresponding analyses could be performed in our case, we would like to emphasize that all hypotheses presented below are purely theoretical and have been deducted from previously published data.

Allo-HSCT is a potential curative treatment option for myeloma patients, but malignant plasma cells often evade the graft-versus myeloma effects. Boosting immunological disease surveillance after allo-HSCT is difficult and often linked to the induction of GvHD [5]. In our patient, treatment response was established by the donor immune system and teclistamab could play a pivotal role in regaining the graft versus myeloma effect, hence providing a long-lasting disease control. In the Majes-TEC1-study, median progression free survival (mPFS) was 11 months and in patients who obtained a complete remission, mPFS was still not reached (at least 27 months) in the latest update [6]. Our patient is now on teclistamab for 3 years with a CR lasting much longer than the data of the Majes-TEC1-study supply. We thus hypothesize, that the enduring treatment effect is mainly driven by the donor immune system and could not be obtained without prior allo-HSCT, but we found no data to further support our presumption. However, it was previously hypothesized that CD8 + effector T cells, which expand after BCMA bispecific antibodies, may possess tumor-reactive properties and interact with MHC molecules on tumor cells [7]. In the post-allo-HSCT setting, the frequency of potentially tumor-reactive T cells could increase, allowing redirection therapies to exert pronounced activity. It would have been of great interest to perform further immunological analyses to support this theory, but the corresponding samples were not available in this patient’s case. Therefore, due to the lack of longitudinal immunophenotyping or T-cell receptor analyses, any discussion regarding potential donor-derived T-cell effects is purely hypothetical. In our patient, complete donor chimerism and absence of prior GvHD point to a stable, tolerized immune environment and teclistamab therapy was initiated more than one year after allo-HSCT, so there was an adequate time for immune reconstitution, which all together might have contributed to the excellent treatment response in our case.

Another recent case report is available for the use of Epcoritamab, an anti-CD20-directed TCE, after allo-HSCT in a case of DLBCL [8]. Intriguingly, Epcoritamab only achieved limited efficacy in this patient, resulting in SD before allo-HSCT. However, re-exposure with Epcoritamab following early progression 2 months after allo-HSCT resulted in an ongoing CR of at least 6 months without any GvHD flare. This case report supports the theory, that TCEs could exert a markedly improved effect after allo-SCT due to an altered composition of the reconstituted donor immune system.

Overall, our presented data may suggest that allo-HSCT followed by TCE therapy in case of relapse represents a potentially promising treatment option for patients for whom other effective therapies, such as CAR-T-cells, are not feasible.

In conclusion, we postulate that in our reported case, allo-HSCT induced a permissive environment for teclistamab-induced long-lasting graft-versus-myeloma response without induction of relevant GvHD. Even though allo-HSCT is not a frequent therapy concept in MM, the subsequent use of TCE is a viable treatment option in case of relapse. The still very limited data at hand indicate that the use of TCE after allo-HSCT could overcome the challenges of T-cell exhaustion in this subset of patients, which so often limits treatment efficacy in heavily pretreated multiple myeloma [9]. Still, further research on this specific constellation would be of interest to provide more data on efficacy, safety and underlying immunological mechanisms of such complex treating algorithms.

## Data Availability

No datasets were generated or analysed during the current study.
